# Real-time detection of Seneca Valley virus by one-tube RPA-CRISPR/Cas12a assay

**DOI:** 10.3389/fcimb.2023.1305222

**Published:** 2024-01-08

**Authors:** Lei Ma, Mengjie Zhu, Qingfeng Meng, Yao Wang, Xueping Wang

**Affiliations:** ^1^ School of Biotechnology and Food Engineering, Anyang Institute of Technology, Anyang, China; ^2^ College of Life Science, Henan University, Kaifeng, China; ^3^ Testing Technology R&D Department, Shanghai Kaiwosha Biotechnology Co., Ltd, Shanghai, China

**Keywords:** Senecavirus A, recombinase polymerase amplification, CRISPR/Cas12a, detection, swine

## Abstract

**Introduction:**

Senecavirus A (SVA) is a highly contagious virus that causes vesicular disease in pigs. At present, laboratory detection methods, such as virus isolation and polymerase chain reaction (PCR), required precision instruments and qualified personnel, making them unsuitable for point-of-care tests (POCT). Fortunately, the emergence of CRISPR/Cas system has provided new opportunities for fast and efficient pathogen detection.

**Methods:**

This study successfully developed a precise and sensitive detection platform for diagnosing SVA by combining the CRISPR system with recombinase polymerase amplification (RPA).

**Results:**

The minimum detection limit of the assay was 10 copies of the SVA genome. Meanwhile, the assay demonstrated high specificity. To validate the effectiveness of this system, we tested 85 swine clinical samples and found that the fluorescence method had a 100% coincidence rate compared to RT-qPCR.

**Discussion:**

Overall, the RPA-CRISPR/Cas12a assay established in our study is a highly effective method for detecting SVA and holds great potential for practical applications in the resource-limited settings.

## Highlights

- A real-time One-Tube RPA-CRISPR/Cas12a assay was established for the detection of Seneca Valley virus.
- The performance of the assay was as good as that of RT-qPCR.
- The assay was a powerful diagnostic platform that offers an alternative to detect SVA in marginalized areas.

## Introduction

Seneca Valley virus (SVV), also known as Senecavirus A (SVA), is the causative pathogen of swine idiopathic vesicular disease (SIVD) (Pasma et al., 2008; Segalés et al., 2017). SVA is a species of ssRNA(+) virus in the family Picornaviridae. SVA was accidentally discovered in PER.C6 cells during the cultivation of adenovirus (Segalés et al., 2017), possibly due to contamination during cell passage. Subsequent retrospective epidemiological surveys have demonstrated that SVA may have spread in American pig herds as early as the late 1980s (Leme et al., 2017). SVA infection has been reported in Canada, the United States, Brazil, and Columbus in 2014 (Leme et al., 2017). SVA was isolated for the first time in China in 2016 (Liu et al., 2020). The clinical signs of SVA infection are characterized by lethargy, anorexia, claudication, and vesicular of the foot and mouth (Leme et al., 2017). The increasing reports of SVA infection have garnered unprecedented attention in the field of veterinary medicine.

The symptoms observed in pigs infected by SVA resemble those caused by vesicular stomatitis virus (VSV) and foot-and-mouth disease virus (FMDV), leading to difficulties in clinical differential diagnosis (Schmitt, 2002; Knight-Jones et al., 2016). Currently, many methods such as PCR-based assays and ELISA have been established for the laboratory diagnosis of SVA (Bracht et al., 2016; Feronato et al., 2018; Mu et al., 2020; Ma Z. et al., 2022; Yan et al., 2023). However, these methods are cumbersome, complex and time-consuming. Furthermore, they require a sophisticated laboratory and professional staff to perform, making it difficult to conduct on-site test experiments for the detection of SVA.

The immune defense system used by prokaryotes to resist foreign virus invasion is known as Clusters of regularly spaced short palindromic repeats (CRISPR) and CRISPR associated protein (Cas protein) (Cong and Zhang, 2015). This system has been transformed into an efficient gene editing tool. Recently, scientists discovered that some class II Cas proteins (such as Cas12a) have accessory cleavage activity and applied the CRISPR-Cas system to the nucleic acid detection field (Chen et al., 2018). The Cas12a protein is distinct from the Cas9 protein. When Cas12a protein binds to the target sequence guided by the gRNA, its ability of arbitrary cutting of all single-stranded DNA is activated. JA Doudna developed a diagnostic system called DNA endonuclease-targeted CRISPR trans reporter (DETECTR) based on the single-stranded DNase activity of the Cas12a protein (Chen et al., 2018). This system can be used to quickly and easily detect infectious pathogens in clinical samples. The DETECTR platform is made up of the recombinant polymerase amplification (RPA), Cas12a protein, CRISPR RNA (CrRNA) and a fluorescent probe. After that, many detection platforms based on RPA and CRISPR were developed for the diagnosis of the infectious diseases caused by various kinds of pathogenic microorganism and parasites (Mayuramart et al., 2021; Sun et al., 2021; Jirawannaporn et al., 2022; Li S. et al., 2022).

**Figure 1:**
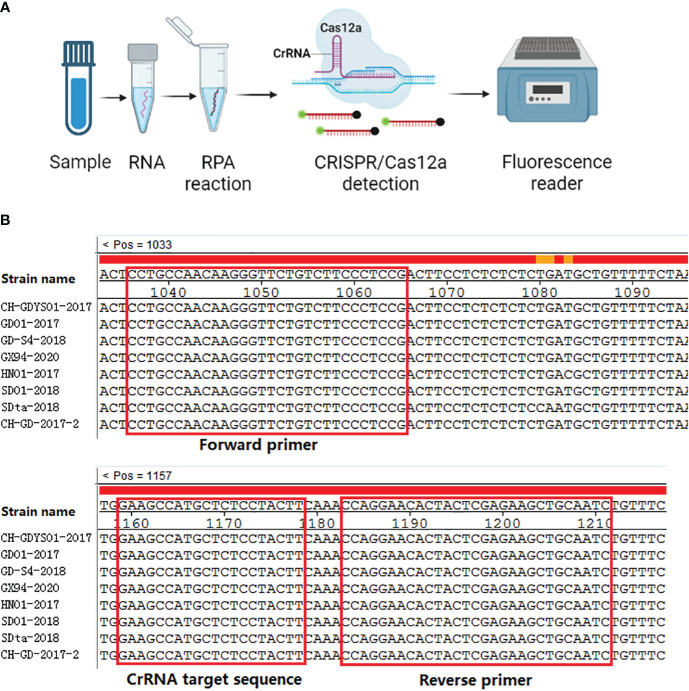
Schematic representation of one-tube RPA-CRISPR/12a assay. **(A)** Workflow of the one-tube RPA-CRISPR/12a assay. **(B)** Sequence alignment of the RPA primers (F3/R3) and CrRNA (CrRNA2). The positions of primers and CrRNA are shown in the red box.

The RPA technology can achieve exponential amplification of template DNA in a short time (20-30 min) and at a constant temperature (39 °C). Currently, there have been several reports about RPA methods for the detection of SVA (Wang et al., 2022a; Wang et al., 2022b). However, CRISPR detection method combined with RPA has not been reported. Thus, our aim is to establish a real-time fluorescence RPA-CRISPR/Cas12a assay for SVA. The RPA-CRISPR/Cas12 detection method will provide a technical basis for rapid diagnosis, prevention and control of SVA. The workflow of the one-tube RPA-CRISPR/12a assay was displayed in **Figure 1A**.

## Materials and methods

### Preparation of the SVA RNA standard

Alignment of the SVA 3D gene were performed and the result demonstrated that the 3D gene was high conserved (**Figure 1B**). Thus, a primers pair (forward primer: 5’-TCTAAATTGAGAAAGACGAC-3’, reverse primer: 5’-ACGGCACCGTAGTCAGCGAA-3’) was designed and synthesized by BGI Co., Ltd (Shenzhen, China) for the amplification of the 3D gene. RT-PCR was performed using the one-step RT-PCR Kit from Takara (Beijing, China) according to the manufacturer’s instruction. After amplification, the RT-PCR product was subjected to 1.0% agarose gel electrophoresis, purified, and cloned into the pGEX-T vector. The positive clone was transcribed using the T7 polymerase Kit from NEB (MA, USA). The transcribed RNA solution was treated with DNase I (ThermoFisher Scientific, MA, USA) and purified using Trizol reagent (Beyotime, Shanghai, China). The concentration of the transcribed RNA was measured using a Nanodrop Microvolume spectrophotometer (ThermoFisher Scientific, MA, USA) and stored at -70°C. The RNA copy number was calculated using a previously reported formula (Ma L. et al., 2022).

### RPA primers design

As of present, no specific software is available for the RPA primer design. However, an internationally recognized RPA products supplier (TwistDx Limited, United Kingdom) provided several guidelines and considerations for RPA design. In our study, we used the online software Primer-BLAST to design the primers. The parameters for the primer design were set according to the guidelines provided by TwistDx.

### RPA primers screening

To assess the amplification efficiency of the RPA primer pairs designed above, basic RT-RPA reactions were conducted separately. The resulting RPA amplicons were then detected by nucleic acid electrophoresis to identify the target band under ultraviolet light.

### RT-RPA reaction

To perform the RT-RPA reaction, a RT-RPA Basic kit (Hangzhou ZC, Hangzhou, China) was utilized according to the manufacturer’s instruction. All the experimental materials were combined in a 0.2 mL reaction tube containing lyophilized preparations, which were placed on ice throughout the procedure. The reaction tubes were promptly sealed and briefly centrifuged. The reaction was initiated on a metal bath at 39 °C for a duration of 20 minutes.

### CrRNA design

The CrRNAs were designed using the CRISPR DT tool available at http://bioinfolab.miamioh.edu/CRISPR-DT/interface/Cpf1_design.php. Each CrRNA sequence consisted of a specific 3D gene sequence and a scaffold sequence. The CrRNA designs can be found in **Table 1**. Additionally, a T7 promoter sequence was added at the beginning of the CrRNA sequence for *in vitro* transcription. The CrRNA sequences were synthesized and inserted into the pGEX-T vector by Sangon (Shanghai, China). Subsequently, the pGEX-T-CrRNA plasmid was transcribed *in vitro* using the method described above.

**Table 1:** The sequences of RPA primers and CrRNAs.

Name	Sequence (5’-3’)
RPA-F1	AATTTGACAGACTGAACAAGGATGTTG
RPA-R1	CAGGATTGCTTCTTTGACGGTCAGAATGT
RPA-F2	TGGACTAGACCCTATGGATCCCCACACAGC
RPA-R2	GAAGACATGATCAGAGTAGTCACCGTCCA
RPA-F3	CCTGCCAACAAGGGTTCTGTCTTCCCTCCG
RPA-R3	GATTGCAGCTTCTCGAGTAGTGTTCCTGG
RPA-F4	CCTGCGCTGGGACCGTATCTCAGATCCCTG
RPA-R4	CTTGTAGGTCAATGCCAGAGCAGTCCTGAT
RPA-F5	AGCGGCCTTCGACGTACTGATCTCGTCGAT
RPA-R5	TTCCCGCCCGGACCTTCTCTGAGGGTCTGA
CrRNA1	UAAUUUCUACUAAGUGUAGAU**GAAAAACAGCAUCAGAGAGA**
CrRNA2	UAAUUUCUACUAAGUGUAGAU**AAGUAGGAGAGCAUGGCUUC**
CrRNA3	UAAUUUCUACUAAGUGUAGAU**CAUCCAUAACUGGUCUAUAU**

Red letters represent the scaffold sequence, bold letters represent the specific 3D gene sequence.

### CrRNA screen

To assess the effectiveness of the CrRNAs in cleaving the target, a CRISPR/Cas12a detection assay was performed. The assay included 2 μl of 10X NEBuffer 2.0 (New England Biolabs, USA), 0.5 μl of Cas12a protein (New England Biolabs, USA), 1 μl of CrRNA (2 μM), 0.5 μl of a single-stranded DNA (ssDNA) probe (5-FAM-TTATTATT-BHQ1-3), and 2 μl of the RT-RPA amplicons. The fluorescent signal resulting from the CRISPR/Cas12a reaction was measured using a Deao instrument (Guangzhou, China) at a temperature of 39°C for 20 minutes. The fluorescence amplitudes were recorded and used to determine the cleavage efficiency of the CrRNAs.

### One-tube RPA-CRISPR/Cas12a assay

The one-step RPA-CRISPR/Cas12a assay involved two steps: the RT-RPA reaction and the Cas12a detection. The RT-RPA assay was performed as the above procedure with a minor modification. A 50 µL RPA reaction mixture was prepared as described above. Additionally, the CRISPR/Cas12a detection reagents (1 µL of DNA FQ Reporter, 1 µL of Cas12a (1.5 µM), 1 µL of CrRNA (1.5 µM), 0.25 µL of RNA inhibitor, and 2 µL of 10X NEBuffer) were applied to the inner surface of the tube lid. Throughout the procedure, the reagents were kept on ice. The tubes were gently covered, incubated at 39°C for 20 minutes, briefly spun down, and then placed into a Deao instrument (Guangzhou, China) at 39°C for 20 minutes to collect the fluorescent signal. The fluorescence was measured for 40 times at 30s internal. If a sample generated an amplification curve above the threshold of the negative control, it was considered positive and below threshold as negative. The results were presented as the time at which the amplification curve begin to appear.

### Optimization of the reaction condition

In the one-step RPA-CRISPR/Cas12a detection method, the specific concentrations of Cas12a protein and CrRNA were of vital important in obtaining the best detection result. To optimize the reaction conditions for the one-step RPA-CRISPR/Cas12a detection method, different concentrations of Cas12a protein and CrRNA were assessed. Various concentrations of CrRNA (2 µM, 1.5 µM, 1 µM, 0.5 µM, 0.25 µM) and Cas12a (2 µM, 1.5 µM, 1 µM, 0.5 µM, 0.25 µM) were tested using the one-step RPA-CRISPR/Cas12a assay. All experiments were performed in triplicate.

### Detection limit of the assay

The 3D RNA standard prepared above was 10-fold diluted using the TE buffer. Seven RNA dilutions (10^6^, 10^5^, 10^4^, 10^3,^ 10^2^, 10^2^, 10 copies/µL and 1 copy/µL) were prepared and used to evaluate the detection limit of the assay. Using the diluted RNA standards as the templates, the RPA-CRISPR/Cas12a assay was carried out, respectively. The test was performed in triplicate. The coefficient of variation (CV) was calculated using the fluorescent intensity values for each RNA standard.

### Specificity

To evaluate the specificity of the assay, several common swine infectious pathogens were tested by the assay. Pig foot-and-mouth disease virus (FMDV), classical swine fever virus (CSFV), porcine reproductive and respiratory syndrome virus (PRRSV), pseudorabies virus (PRV), porcine rotavirus (RV), porcine circovirus type 2 (PCV2), and pseudorabies virus (PRV) were included in this study. The specific information of these viruses were described in a previous study (Ma L. et al., 2022). The nucleic acid extracted from SVA clinical sample was used as a positive control. The test was performed in triplicate.

### The performance of the assay on clinical samples

The feasibility of the RPA-CRISPR/Cas12a assay for detecting SVA was assessed using 85 clinical specimens. These swine specimens were collected from pig tissues with vesicular lesions in Henan Province between April 2019 and February 2023. These specimen were collected for laboratory diagnosis, and not specifically for our study. Furthermore, no animals were experimentally infected in our study; no IACUC protocols apply or are available. Tissue homogenate was prepared as a 10% (w/v) suspension using Minimum Essential Medium (ThermoFisher Scientific, MA, USA) and clarified by centrifugation for 10 min at 12000r/s. The supernatant was collected. The nucleic acids of the samples were isolated by RNAeasy™ Animal RNA Extraction Kit (Beyotime, Shanghai, China) and tested by the RPA-CRISPR/Cas12a assay. The samples were also analyzed by a RT-qPCR assay targeting the capsid protein gene according to the China Agricultural industry standard (NY/T 3790-2020). In both of these detection methods, positive and negative controls were set up separately. Prism 5.0 software (GraphPad) was used to analyze the correlation between RPA-CRISPR/Cas12a threshold time (TT) and RT-qPCR cycle threshold (Ct) values.

## Results

### Primer screen

**Figure 2:**
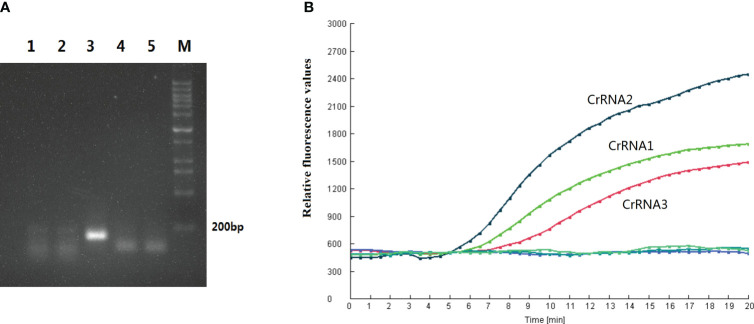
Primer and CrRNA assessment. **(A)** Five RPA primer pairs were designed. Basic RT-RPA assay was carried out to screen the best primers. **(B)** Evaluation the effect of the CrRNAs by the Cas12a detection assay.

In this study, five RPA primer pairs were designed. A basic RT-RPA assay was carried out to screen the best primers. RPA product of F3/R3 produced a single band with a strong signal under UV light, and the intensity of the band was greater than the other four primer sets (**Figure 2A**). Therefore, the F3/R3 primers were used for the RPA assay.

### CrRNA screen

In this study, three CrRNAs were designed. To determine the effect of the CrRNAs, the RPA product of F3/R3 was detected by Cas12a detection assay. As illustrated in **Figure 2B**, the fluorescent signals of all CrRNAs had a continuous rise over time. The negative control generated no fluorescence signal throughout the whole reaction process. Meanwhile, there was a difference in the activity of three CrRNAs, with CrRNA2 showing the greatest efficiency because of the high fluorescence intensity and short reaction time. A remarkable increase of the fluorescent signal was observed at 5 min, which was quicker than the other two CrRNAs. Moreover, the intensity of the fluorescent signal was strongest among the three CrRNAs. Thus, CrRNA2 was used for the subsequent one-step RPA-CRISPR/Cas12a assay.

### Reaction conditions

**Figure 3:**
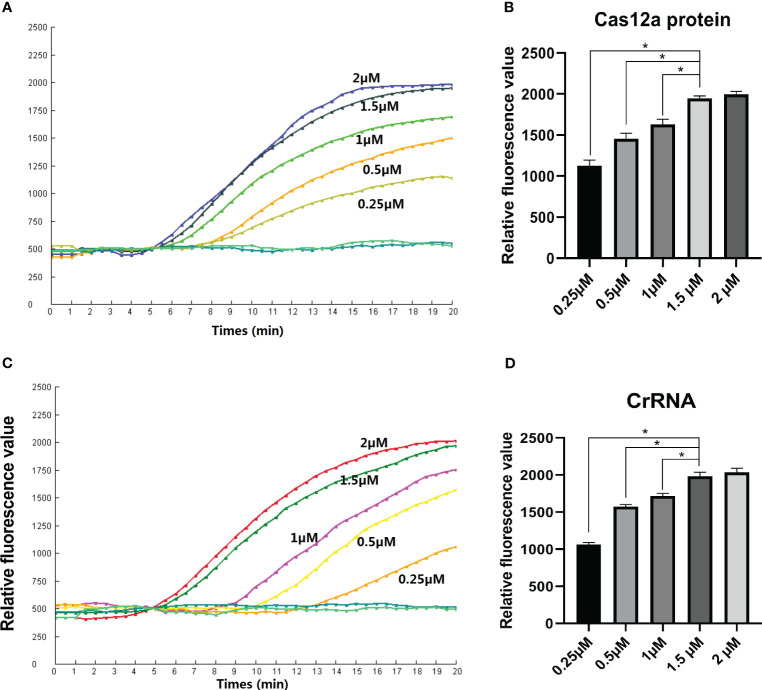
Optimization of the Reaction conditions. **(A, B)** Cas12a concentrations (2 µM, 1.5 µM, 1 µM, 0.5 µM, 0.25 µM) were assessed; **(C, D)** Different CrRNA concentrations (2 µM, 1.5 µM, 1µM, 0.5µM, 0.25µM) were assessed. The test was performed in triplicate.

Various concentrations of CrRNA and Cas12a were adjusted in order to obtain the best detection result for the assay. The results indicated that increasing the Cas12a concentration (1µM, 0.5µM, 0.25µM) led to an increase in the fluorescence intensity peak. However, the peaks of the fluorescence intensity of different Cas12a concentrations (2 µM, 1.5 µM) did not have many differences. Hence, the Cas12a concentration of 1.5 µM was selected for the following assay (**Figure 3A**). Next, the optimal concentration of CrRNA was investigated. As illustrated in **Figure 3B**, when a higher concentration of CrRNA (1 µM, 0.5 µM, 0.25 µM) was used, the assay took a shorter time to achieve the exponential amplification. As the CrRNA concentration was more than 1 µM, the time of achieving the exponential amplification and the fluorescence intensity didn’t have much difference. Therefore, CrRNA at the concentration of 1.5 µM was used in the RPA-CRISPR/Cas12a assay (**Figures 3C, D**).

### Sensitivity

**Figure 4:**
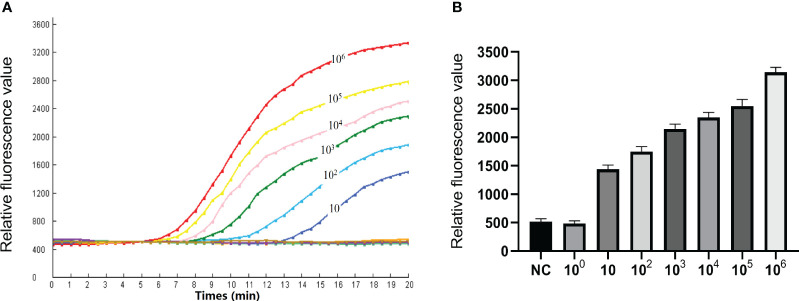
Sensitivity of the RPA-CRISPR/Cas12a assay. **(A)** To determine the minimum detection of the RPA CRISPR/Cas12a assay, ten-fold dilution standards were detected as templates by the method. **(B)** The test was performed in triplicate.

To assess the detection limit of the RPA-CRISPR/Cas12a method, we conducted experiments using ten-fold dilution standards as templates. These RNA standards (10^6^-10 copies) tested positive for SVA. Meanwhile, RNA dilution containing 1 copy gene tested negative for SVA. The fluorescence intensity of three negative control did not increase throughout the whole reaction process (**Figures 4A, B**). These results clearly demonstrate that the real-time RPA-CRISPR/Cas12a method is capable of detecting as few as 10 copies of the SVA RNA genome. The assay repeatability was assessed by CV. The CVs were 2.6%, 3.5%, 3.3%, 2.0%, 6.1% and 3.4% for the RNA standards (10^6^-10 copies) respectively.

### Specificity

**Figure 5:**
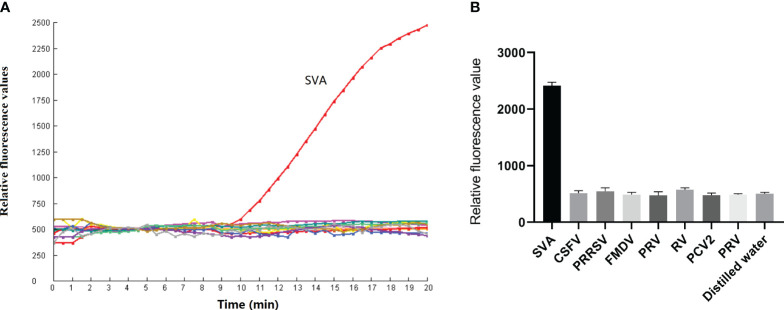
Specificity of the RPA-CRISPR/Cas12a assay. **(A)** CSFV, PRRSV, FMDV, PRV, RV, PCV2 and PRV were detected by the RPA-CRISPR/Cas12a assay. **(B)** The test was performed in triplicate.

The fluorescence intensity of the SVA-positive clinical sample increased significantly after a 10 min reaction time. The distilled water was tested negative by the RPA-CRISPR/Cas12a assay. The result showed the test result was valid. Meanwhile, CSFV, PRRSV, FMDV, PRV, RV, PCV2 and PRV tested negative by the RPA-CRISPR/Cas12 assay (**Figures 5A, B**). These findings confirm that the RPA-CRISPR/Cas12a method is highly specific and can accurately diagnose SVA without cross-reactivity to other pathogens.

### Clinical performance of the assay

**Figure 6:**
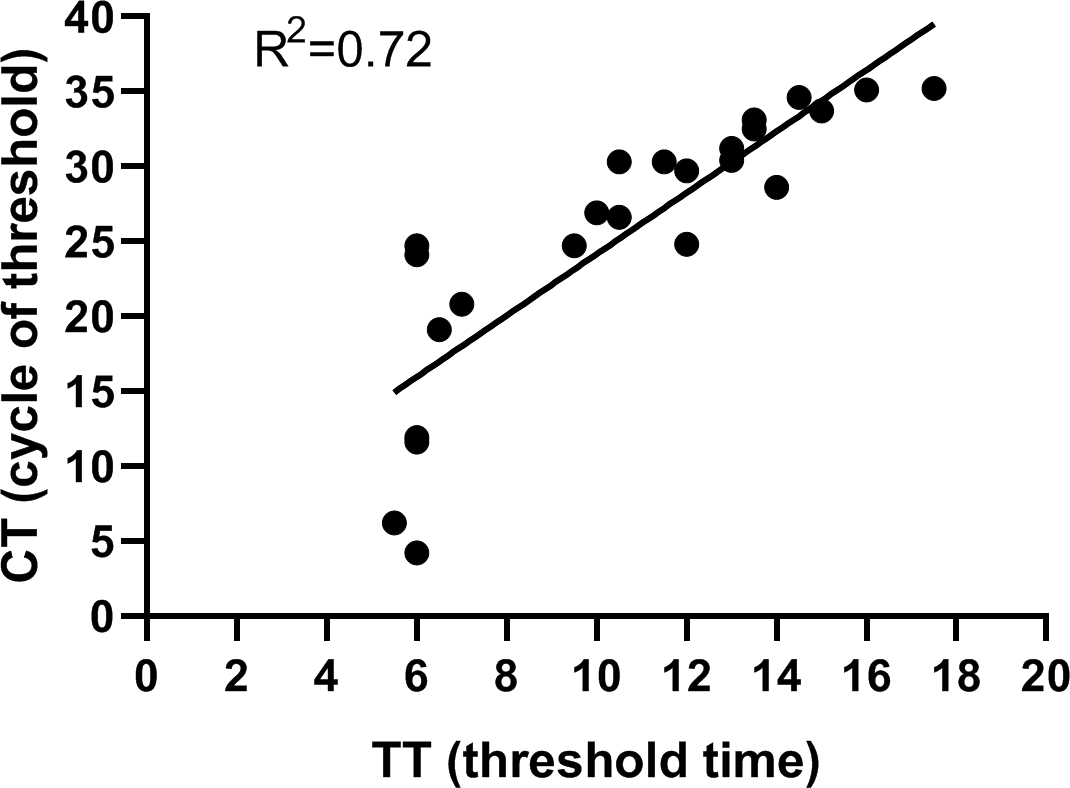
Performance of the RPA-CRISPR/Cas12a assay. Linear regression analysis of RPA-CRISPR/Cas12a threshold time (TT, x axis) and RT-qPCR cycle threshold values (CT, y, axis) were determined by Prism software. R^2^ value was 0.72.

To validate the performance of the assay established in our study, a total of 85 clinical samples were tested. Of the 85 samples, 23 samples tested positive and 62 tested negative by the RPA-CRISPR/Cas12a assay. Meanwhile, 23 tested positive and 62 tested negative by RT-qPCR assay. Furthermore, all the samples found to be positive for SVA by the RPA-CRISPR/Cas12a method were also positive when tested with RT-qPCR. All the SVA-negative samples determined by RPA-CRISPR/Cas12a were also negative by RT-qPCR. The agreement between the two methods was 100%, indicating that RPA-CRISPR/Cas12 established in the present study had robust detection capability for clinical samples (**Table 2**). The linear regression analysis indicated a weak correlation between TT values and CT values, suggesting that the RPA-CRISPR/Cas12a assay is not suitable for quantitative detection of SVA (**Figure 6**). The raw data for the 85 clinical samples was provided in supplements.

**Table 2:** Comparison of RPA-CRISPR/Cas12a and RT-qPCR.

			RT-qPCR		CR
		Positive	Negative	Total	
RPA-CRISPR/Cas12a	Positive	23	0	23	
	Negative	0	62	65	100%
	Total	23	62	85	

CR, coincidence rate. CR = (23 + 62)/85*100%.

## Discussions

SVA was responsible for the presence of vesicular lesions on the tongue and feet of the infected pig. However, other viruses such as FMDV and SVDV can also cause similar clinical signs as SVA. To accurately diagnose SVA infection, it is important to have a fast and reliable diagnostic method. In this study, we successfully developed a real-time RPA-CRISPR/Cas12a detection method to detect SVA.

In the last 10 years, the short reaction time and high sensitivity of the RPA method have promoted its rapid development (Li et al., 2018; Tan et al., 2022). To date, more than one hundred detection methods based on RPA technology have been established for the diagnosis of various kinds of infectious pathogens (Ma et al., 2019; Ma et al., 2020; Ma L. et al., 2022; Zhu et al., 2022). The RPA assay is currently the fastest among molecular detection methods. However, RPA also has similar issues as other nucleic acid amplification method, such as non-specific amplification. In this regard, CRISPR technology has emerged as a promising solution for rapid diagnostics (Chen et al., 2018). Among the Cas proteins, the Cas12a protein is highly valued for its ability to recognize and cleave DNA with high specificity. The Cas12a’s non-specific DNase activity can be specifically activated in the presence of a specified CrRNA, which allows it to cleave the ssDNA probe used for diagnostic purposes. Several One-Pot platforms utilizing RPA-CRISPR/Cas12a assay have been developed and applied for the effective, rapid, and sensitive detection of various pathogens in infectious disease diagnosis (Aman et al., 2020; Dai et al., 2022; Jirawannaporn et al., 2022; Li Y. et al., 2022; Verma et al., 2022; Hu et al., 2023).

In our study, we established a rapid fluorescence detection assay based on RPA and Cas12a technology. We used the method to detect the 3D gene and identify the presence of SVA in clinical specimens. The entire experiment took approximately 60 minutes to complete without requiring sophisticated equipment.

The real-time RPA-CRISPR/Cas12a method was an integration of RPA reaction and Cas12a detection. Two key aspects to consider in the development of this method were the design of primers for RPA reaction and CrRNA design for Cas12a detection. Unlike PCR and qPCR, there is no specific software available, such as Oligo or Primer Premier, to assist in RPA primer design, making it more challenging. Nevertheless, RPA Commercial Development Company (TwistDX) provides relevant suggestions on primer design: the amplification product should be within 500 bp, ensuring the absence of repetitive nucleotide sequences, maintaining a GC content between 30% and 70%, and preventing primer dimer and secondary structure formation. In our study, we designed five sets of primers following the aforementioned recommendations. The primer set F3/R3 showed the best amplification effect. Multiple studies have investigated different reaction temperatures and times (Zhang et al., 2020; Ma L. et al., 2022), and it has been demonstrated that a reaction temperature of 39°C and a reaction time of 20 minutes yield optimal results for the RPA assay (Jirawannaporn et al., 2022; Li Y. et al., 2022; Hu et al., 2023). Thus, the reaction conditions were also used in our study for the RPA reaction.

The CrRNA sequence contained a Cas12a protospacer adjacent motif (PAM) sequence (5-TTTV). The PAM sequence should be included in the gene region amplified by the RPA primer F3/R3. Three specific CrRNAs are designed based on the target sequence of the F3/R3 primers. The cleavage activities of the CrRNAs were tested. The result showed that one CrRNA possessed the best cleaved capacity among the three CrRNA.

The Cas12a protein and RPA reaction were generally conducted in two steps. Previous studies have typically performed the RPA reaction first, followed by testing the RPA product with Cas12a (Qian et al., 2021; Zhang et al., 2022). However, this after-amplification procedure may lead to aerosol contamination and non-specific amplification. In our study, we adopted a one-step procedure that had been successfully established in a RPA-CRISPR/Cas12 study (Lin et al., 2022). The one-step RPA-CRISPR/Cas12a method demonstrated outstanding ability for detecting SVA in clinical samples. The assay developed in our study reduced the experimental procedure and required less time compared to the two-step RPA assays.

In conclusion, we successfully developed a real-time RPA-CRISPR/Cas12a method for detecting SVA. This diagnostic approach can detect 10 copies of the SVA genome per reaction and does not cross-react with other common swine pathogens. It is a specific, sensitive, and time-efficient method. Furthermore, it enables effective detection of SVA in clinical samples. The performance of the RPA-CRISPR/Cas12a assay is comparable to that of RT-qPCR. This method is highly suitable for veterinary diagnostic laboratories and field detection on farms, facilitating rapid diagnosis at the early stage of vesicular clinical symptoms in pigs and enabling timely and effective control of disease spread.

## Data availability statement

The raw data supporting the conclusions of this article will be made available by the authors, without undue reservation.

## Author contributions

LM: Conceptualization, Formal Analysis, Writing – original draft. MZ: Writing – original draft, Writing – review & editing. QM: Supervision, Writing – review & editing. YW: Writing – review & editing. XW: Supervision, Validation, Writing – review & editing.
