# Supplementary material for: Real-time detection of Seneca Valley virus by one-tube RPA-CRISPR/Cas12a assay
Source: Front Cell Infect Microbiol. 2024 Jan 8;13:1305222. doi: 10.3389/fcimb.2023.1305222 (PMC10800940; doi:10.3389/fcimb.2023.1305222)

Supplements. Fig.1 Detection results of the RPA-CRISPR/Cas12a method. A positive and a negative control were included.

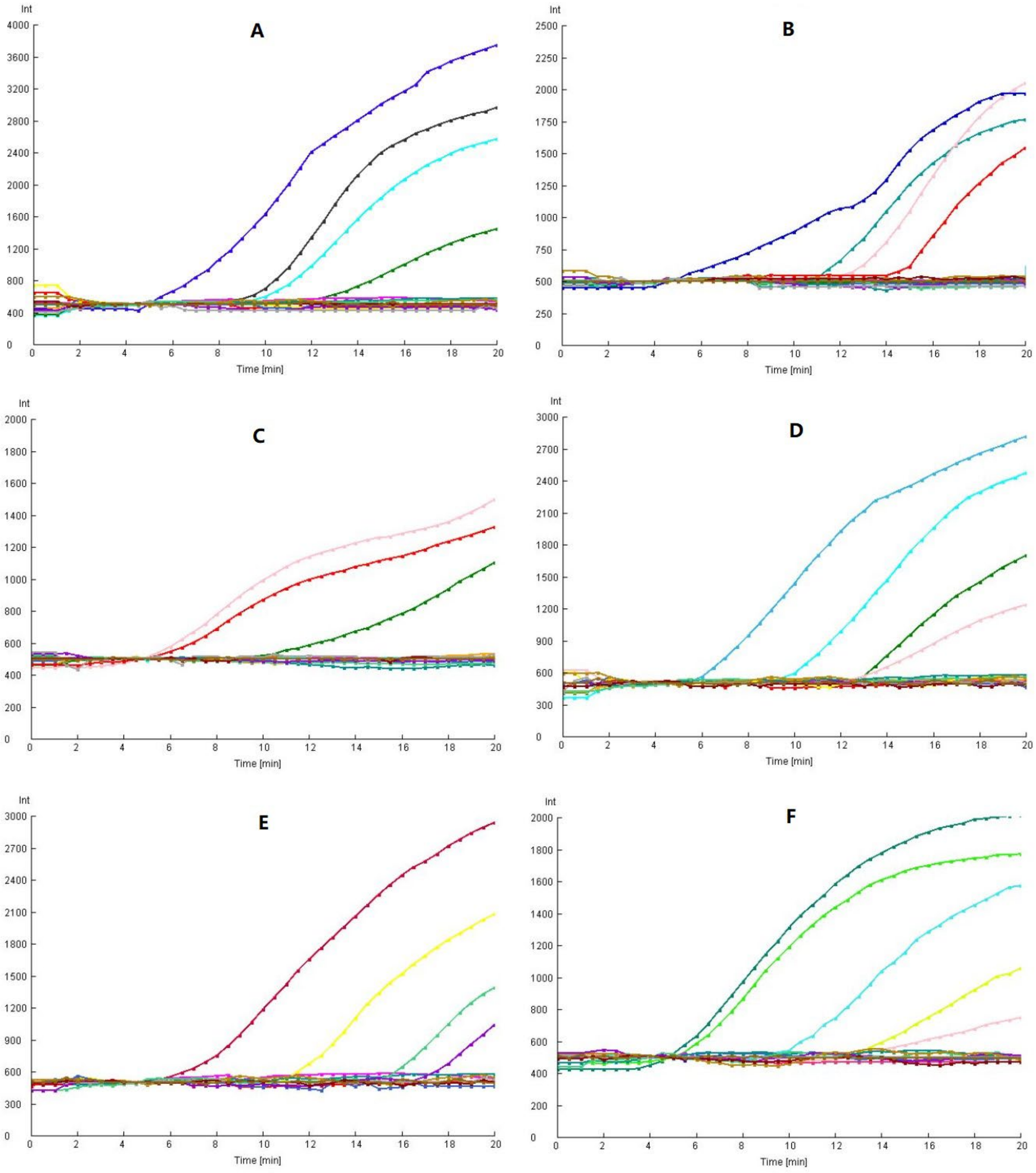

Supplements. Fig.2 Detection results of the RT-qPCR method. A positive and a negative control were included.

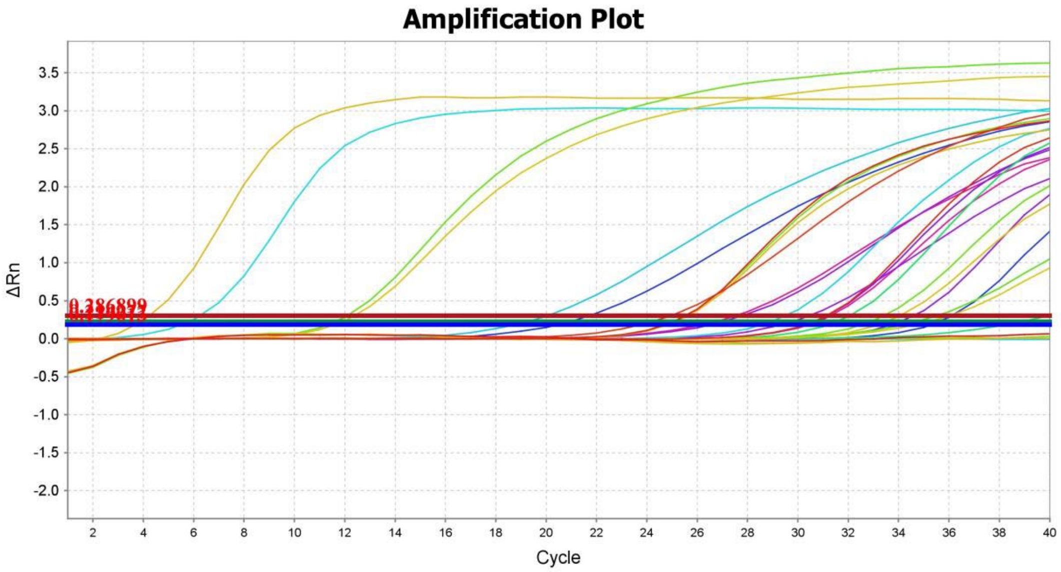

Supplement: Supplementary file 1 [file Image_1.pdf]
